# Epigenetic modulation of thyroid cancer metastasis and glycolysis through circSSU72-mediated ubiquitination of gamma-catenin and beta-catenin signaling

**DOI:** 10.1016/j.gendis.2024.101485

**Published:** 2024-12-07

**Authors:** Zeyu Zhang, Duntao Su, Xiangyuan Qiu, Lei Yao, Fada Xia, Xinying Li

**Affiliations:** a Department of Thyroid Surgery, Xiangya Hospital, Central South University, Changsha, Hunan 410000, China; b National Clinical Research Center for Geriatric Disorders, Xiangya Hospital, Central South University, Changsha, Hunan 410000, China; c Department of General Surgery, Xiangya Hospital, Central South University, Changsha, Hunan 410000, China

The incidence of thyroid cancer (TC) has continuously risen worldwide in the past three decades. While most TC patients have a good prognosis, 60% of them still suffer from lymph node metastasis, which is significantly associated with patient prognosis.^1^ Circular RNAs (circRNAs) are a large class of noncoding RNAs that function as tumor suppressors or tumor promoters in multiple human cancers, including TC.^2^ In the previous study, we found one circRNA (circSSU72) was significantly up-regulated in both tissues and cell lines of papillary thyroid carcinoma.^3^ However, the biological role and associated mechanisms of circSSU72 in TC, especially in the field of metastasis, have not been well elucidated.

In the present study, our study demonstrated that circSSU72 could bind to the armadillo (ARM) domain of junction plakoglobin (JUP, also known as gamma-catenin) and promote K48-specific ubiquitination by enhancing the binding between JUP and retinoblastoma-binding protein 6 (RBBP6), influencing desmosomes and the beta-catenin pathway and thus promoting TC metastasis and glycolysis. m^6^A modification at position 546 might involve the up-regulated expression of circSSU72. These findings highlighted the circSSU72/JUP axis as a potential diagnostic marker and a therapeutic target in TC. This study was approved by the Institutional Ethics Committee of Xiangya Hospital, Central South University (No. 201908226), and all subjects signed the written consent.

circSSU72 (hsa_circ_0009294) was derived from two exons of the SSU72 gene, containing 614 nucleotides (Fig. S1A). First, we reconfirmed circSSU72 level was significantly up-regulated in TC (Fig. 1A), which was consistent with TC cell lines (Fig. S1B), and associated with worse tumor characteristics (Table S1). Compared with linear SSU72 mRNA, circSSU72 showed significant insensitivity to RNase R and actinomycin *D* (Fig. S1C, D). Nuclear and cytoplasmic RNA separation (Fig. S1E) showed that circSSU72 was mainly localized in the cytoplasm. After the effective knockdown and overexpression of circSSU72 (Fig. S1F), the CCK-8 assays, colony formation assays, wound healing assays, and Transwell assays showed that circSSU72 could significantly promote TC cell proliferation, migration, and invasion (Fig. S1G–N).

**Figure 1:**
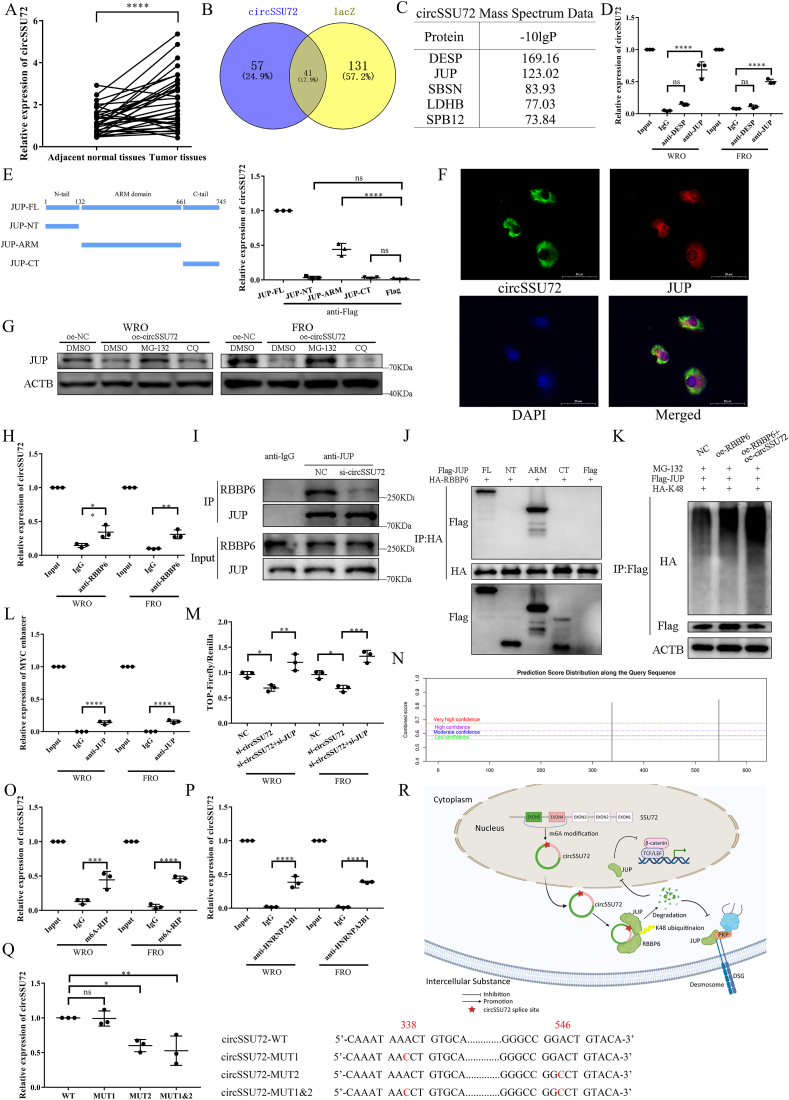
N6-methyladenosine-mediated circSSU72 promotes metastasis and glycolysis in thyroid cancer by facilitating RBBP6-induced ubiquitination of JUP. **(A)** The expression of circSSU72 in thyroid cancer tissues (*n* = 32). **(B, C)** Venn diagram and proteins identified with the chromatin isolation by RNA purification and mass spectrometry. **(D)** The interaction between JUP and circSSU72 was reconfirmed by RNA immunoprecipitation assays (*n* = 3). **(E)** RNA immunoprecipitation assay showed that circSSU72 interacted with the ARM domain of JUP in HEK293T cells (*n* = 3). **(F)** Fluorescence in situ hybridization and immunofluorescence assays showed circSSU72 and JUP were co-localized in the cytoplasm. **(G)** The circSSU72-induced degradation of JUP could be rescued by 15 μM MG-132 (a proteasome inhibitor) but not by 20 μM chloroquine (a lysosome inhibitor). **(H)** The interaction between RBBP6 and circSSU72 was reconfirmed by RNA immunoprecipitation assay (*n* = 3). **(I)** Knockdown of circSSU72 reduced the binding between RBBP6 and JUP. **(J)** RBBP6 interacted with the ARM domain of JUP in HEK293T cells. **(K)** circSSU72 affected K48-specific ubiquitination of JUP in HEK293T cells. **(L)** Chromatin immunoprecipitation assays showed that JUP could bind to the MYC enhancer (a typical binding site for the TCF/LEF transcription factor). **(M)** The TOP-luciferase assay showed that the circSSU72/JUP axis could influence the transcription of TCF/LEF-DNA complexes. **(N)** The m^6^A modification site of circSSU72 was predicted by the SRAMP database. **(O)** Methylated RNA immunoprecipitation showed that circSSU72 was associated with m^6^A modification (*n* = 3). **(P)** RNA immunoprecipitation reconfirmed the interaction between circSSU72 and HNRNPA2B1 (*n* = 3). **(Q)** RNA immunoprecipitation of HNRNPA2B1 in HEK293T cells showed that position 546 (GGACT) was an essential motif in m^6^A modification of circSSU72 (*n* = 3). **(R)** Graphical illustration of the regulatory mechanisms of the circSSU72/JUP axis in thyroid cancer. One-way analysis of variance (ANOVA) and Welch's ANOVA were applied for quantitative analyses. ∗*p* < 0.05; ∗∗*p* < 0.01; ∗∗∗*p* < 0.001; ∗∗∗∗*p* < 0.0001.

Subsequently, we identified 57 proteins that specifically bound to circSSU72 with chromatin isolation by RNA purification and mass spectrometry (Fig. 1B). The two most significant proteins (DESP and JUP) were chosen for further validation, while only the binding between circSSU72 and JUP was reconfirmed (Fig. 1C, D). Meanwhile, there was no significant binding between the linear SSU72 gene and the two proteins (Fig. S2A). Further RNA immunoprecipitation (RIP) assays in circSSU72-overexpressing HEK293T cells showed that circSSU72 interacted with the ARM domain of JUP (Fig. 1E). JUP was down-regulated in TC tissues compared with adjacent normal thyroid tissues, and TC tissues in the circSSU72-high group showed a significantly lower expression of JUP (*n* = 32; Fig. S2B), while the low JUP level was associated with lymph node metastasis (Table S2). After the knockdown or overexpression of circSSU72 in TC cells, the protein level of JUP changed accordingly, while the mRNA level of JUP did not (Fig. S3A, B), indicating that the regulation of JUP by circSSU72 might occur at the posttranslational level. The fluorescence in situ hybridization and immunofluorescence assays showed circSSU72 and JUP were co-localized in the cytoplasm (Fig. 1F).

We then examined JUP protein stability with cycloheximide, showing that circSSU72 significantly destabilized the JUP protein (Fig. S3C). Moreover, MG-132 partially reversed the regulatory effect of circSSU72 on JUP, while chloroquine did not (Fig. 1G). Meanwhile, the knockdown of circSSU72 could inhibit the ubiquitination of JUP (Fig. S3D). These results indicated that the regulatory effect of circSSU72 on JUP might involve the ubiquitin-proteasome system. Among 57 circSSU72-specific binding proteins, we noticed an E3 ubiquitin-protein ligase, RBBP6. Thus, we assumed that circSSU72 might play a role in the binding between RBBP6 and JUP. RIP assay confirmed the binding between circSSU72 and RBBP6 (Fig. 1H), while co-immunoprecipitation assays showed that RBBP6 could bind to JUP (Fig. S3E). Meanwhile, the knockdown of RBBP6 increased the expression of JUP by inhibiting JUP ubiquitination (Fig. S3F, G). Further, co-immunoprecipitation assays showed that the knockdown of circSSU72 could reduce the binding between RBBP6 and JUP (Fig. 1I). Consistently, the knockdown of circSSU72 rescued the RBBP6-induced ubiquitination of JUP (Fig. S3H, I). Further investigations showed that RBBP6 interacted with the ARM domain of JUP (Fig. 1J). To investigate the specific ubiquitination type of JUP, K48 and K63 ubiquitin were transfected with Flag-JUP in HEK293T cells. The results showed that circSSU72 affected the K48-specific ubiquitination of JUP (Fig. 1K; Fig. S3J). These results suggested that circSSU72 promoted the binding between RBBP6 and the ARM domain of JUP, thus affecting the K48-specific ubiquitination of JUP.

After the knockdown of JUP (Fig. S4A, B), CCK-8 assays, colony formation assays, wound healing assays, and Transwell assays revealed that the knockdown of JUP could significantly rescue the effects of circSSU72 knockdown on TC cell proliferation, migration, and invasion (Figs. S4C–J). JUP is reported to play an essential role in desmosomes.^4^ Thus, we investigated the role of the circSSU72/JUP axis in desmosomes. A hanging-drop assay, desmosome-related protein level, and transmission electron microscopy showed that regulation of circSSU72 significantly affected the cell–cell adhesion and desmosomes, while regulation of JUP significantly rescued these effects (Fig. S5A–C), indicating a role of circSSU72/JUP axis in desmosome structure of TC cells.

Besides, we also found the circSSU72/JUP axis functioned via the beta-catenin signaling pathway (Fig. S6A). Interestingly, while the expression of nuclear JUP was regulated accordingly, the expression of nuclear beta-catenin remained unchanged (Fig. S6B). Subsequent chromatin immunoprecipitation assays showed that JUP could bind to the MYC enhancer, a typical binding site for the TCF/LEF transcription factor (Fig. 1L), indicating binding between JUP and TCF/LEF-DNA complexes. Moreover, the TOP-luciferase assay showed that the circSSU72/JUP axis could influence the transcription of TCF/LEF-DNA complexes (Fig. 1M). PNU-74654, a known beta-catenin signaling pathway inhibitor, was applied to confirm the effect of the circSSU72/JUP axis on the pathway (Fig. S7A). The CCK-8 assays, colony formation assays, wound healing assays, and Transwell assays (Figs. S7B–I) revealed that the effects of the circSSU72/JUP axis on TC cell proliferation, migration, and invasion could be inhibited by PNU-74654. In the pathway analysis of circSSU72-specific binding proteins, we noticed the involvement of glucose metabolism (Supplement Material 1), in which the Wnt/beta-catenin signaling pathway is reported to be involved.^5^ The knockdown of circSSU72 led to a significantly decreased level of glucose uptake, lactate production, ATP level, and extracellular acidification rate, while the knockdown of JUP could significantly rescue these effects (Fig. S7J–Q).

To validate the effect of the circSSU72/JUP axis on TC metastasis, we established metastatic models *in vivo*. The results showed that circSSU72 could significantly promote TC cell metastasis, which was reversed by JUP (Fig. S8A–C). Moreover, the Western blot results and transmission electron microscopy confirmed the effects of the circSSU72/JUP axis on desmosomes and the beta-catenin signaling pathway (Fig. S8D, E).

An additional endeavor was made to investigate the reasons for the dysregulated expression of circSSU72 in TC. In all 98 circSSU72-binding proteins, we noticed HNRNPA2B1, an m^6^A reader, indicating that m^6^A modification might be involved. The SRAMP database predicted that there were two potential sites of m^6^A modification in circSSU72 with very high confidence (Fig. 1N). Methylated RIP assays confirmed that circSSU72 was involved in m^6^A modification (Fig. 1O). Further, RIP assays showed that there was binding between circSSU72 and HNRNPA2B1 (Fig. 1P). Interestingly, we also found binding between linear SSU72 mRNA and HNRNPA2B1 (Fig. S9A). Data on thyroid cancer from the TCGA database showed that HNRNPA2B1 was significantly down-regulated and associated with a better tumor stage (Fig. S9B, C). The knockdown of circSSU72 showed no effect on the HNRNPA2B1 protein level (Fig. S9D). However, the knockdown of HNRNPA2B1 significantly increased the circSSU72 level (Fig. S9E, F). To investigate the specific m^6^A motif, we created three mutant plasmids according to the predicted m^6^A site. RIP assays showed that position 546 (GGACT) was an essential motif (Fig. 1Q). These results indicated that m^6^A modification involved the up-regulated level of circSSU72 in TC (Fig. 1R).

## Ethics declaration

This study was approved by the Institutional Ethics Committee of Xiangya Hospital, Central South University (No. 201908226), and all subjects signed the written consent. This study strictly complied with the Declaration of Helsinki and its later amendments or comparable ethical standards.

## Funding

This work was supported by the 10.13039/501100001809National Natural Science Foundation of China (No. 82073262).

## CRediT authorship contribution statement

**Zeyu Zhang:** Conceptualization, Data curation, Formal analysis, Methodology, Project administration, Visualization, Writing – original draft. **Duntao Su:** Formal analysis, Validation, Writing – review & editing. **Xiangyuan Qiu:** Formal analysis, Validation, Writing – original draft. **Lei Yao:** Methodology, Writing – review & editing. **Fada Xia:** Investigation, Methodology, Software, Writing – original draft. **Xinying Li:** Conceptualization, Funding acquisition, Supervision, Writing – review & editing.

## Data availability

The datasets supporting the conclusions of this article are included within the article and its additional files.

## Conflict of interests

The authors declared no competing interests.

## Figures and Tables

**Figure S1:**
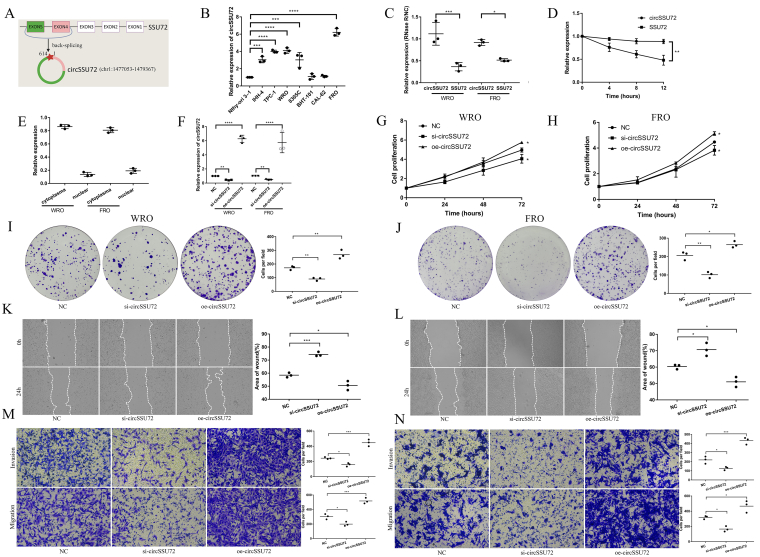
circSSU72 was up-regulated in thyroid cancer. **(A)** The diagram exhibiting the formation of circSSU72 (hsa_circ_0009294). **(B)** The expression of circSSU72 in normal thyroid and thyroid cancer cell lines (*n* = 3). **(C)** The relative expression of circSSU72 and SSU72 mRNA after treatment with RNase R (*n* = 3). **(D)** The relative expression of circSSU72 and SSU72 mRNA after treatment with actinomycin *D* (*n* = 3). **(E)** circSSU72 was separately detected in the nucleus and cytoplasm (*n* = 3). **(F)** Verification of siRNA and vector transfection in thyroid cancer cell lines. **(G, H)** CCK-8 assay for WRO and FRO cells. **(I, J)** Representative images and quantitative analysis of colony formation in WRO and FRO cells. **(K, L)** Representative images and quantitative analysis of the wound healing assay for WRO and FRO cells. **(M, N)** Representative images and quantitative analysis of the Transwell assay for WRO and FRO cells. One-way analysis of variance (ANOVA) and Welch's ANOVA were applied for quantitative analyses. The results shown are representative of three independent experiments. ∗*P* < 0.05; ∗∗*P* < 0.01; ∗∗∗*P* < 0.001; ∗∗∗∗*P* < 0.0001.

**Figure S2:**
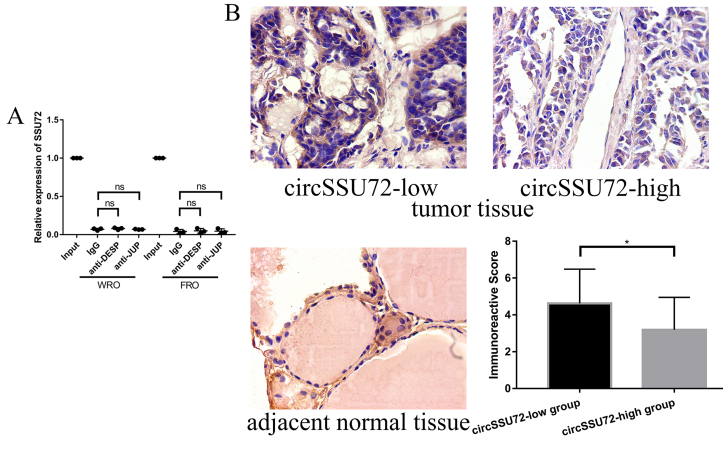
The expression level of JUP in thyroid cancer tissues. **(A)** No interaction was shown between JUP and linear SSU72 mRNA by RNA immunoprecipitation assay (*n* = 3). **(B)** Representative immunohistochemistry staining images of JUP in normal thyroid and thyroid cancer tissues (*n* = 32). One-way analysis of variance (ANOVA) and Welch's t-test were applied for quantitative analyses. ∗*P* < 0.05; ∗∗*P* < 0.01; ∗∗∗*P* < 0.001; ∗∗∗∗*P* < 0.0001.

**Figure S3:**
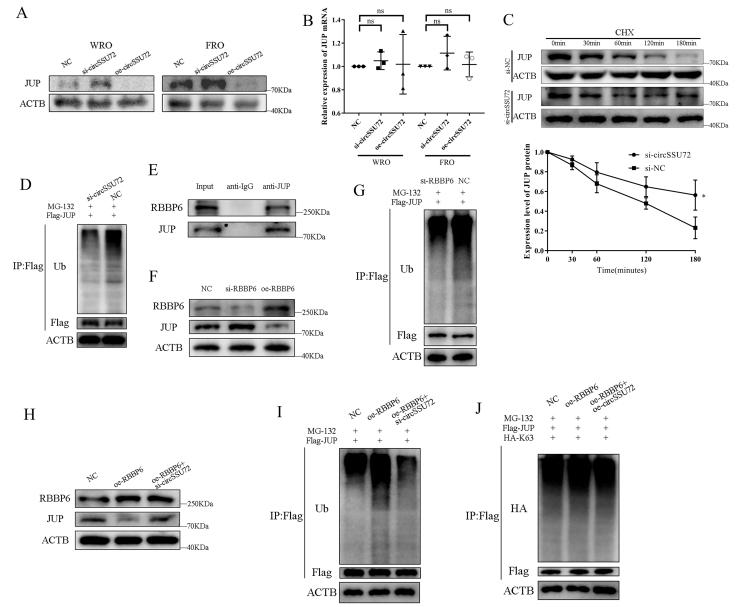
The knockdown of circSSU72 reduced RBBP6-induced K48-specific ubiquitination of JUP. **(A)** The expression of JUP was regulated by circSSU72. **(B)** The expression of JUP mRNA was not influenced by circSSU72 (*n* = 3). **(C)** WRO cells were treated with cycloheximide (150 μM) to investigate JUP protein stability with circSSU72 knockdown. **(D)** Ubiquitination of JUP was determined by immunoprecipitation and western blot assays. **(E)** The interaction between RBBP6 and JUP was confirmed with co-immunoprecipitation assays. **(F, G)** RBBP6 promoted the ubiquitination degradation of JUP. **(H, I)** The knockdown of circSSU72 rescued the RBBP6-induced ubiquitination of JUP. **(J)** circSSU72 did not affect the K63-specific ubiquitination of JUP in HEK293T cells. One-way analysis of variance (ANOVA) and Welch's *t*-test were applied for quantitative analyses. The results shown are representative of three independent experiments. ∗*P* < 0.05; ∗∗*P* < 0.01; ∗∗∗*P* < 0.001; ∗∗∗∗*P* < 0.0001.

**Figure S4:**
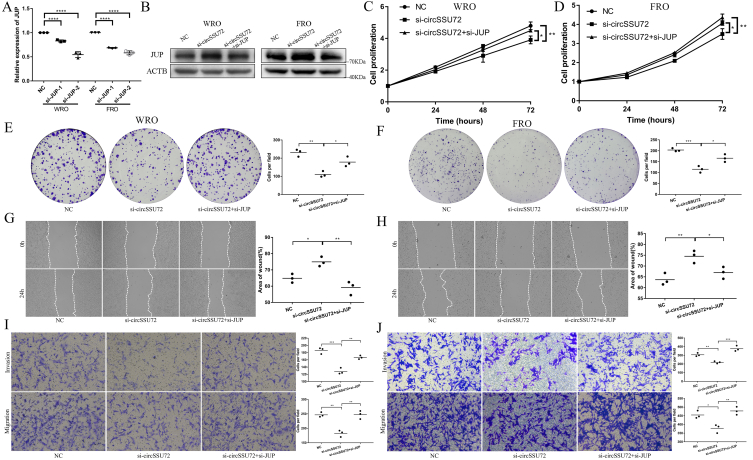
circSSU72/JUP axis affected thyroid cancer cell proliferation, migration, and invasion. **(A, B)** Verification of siRNA transfection in thyroid cancer cell lines (*n* = 3). **(C, D)** CCK-8 assay for WRO and FRO cells. **(E, F)** Representative images and quantitative analysis of colony formation in WRO and FRO cells. **(G, H)** Representative images and quantitative analysis of the wound healing assay for WRO and FRO cells. **(I, J)** Representative images and quantitative analysis of the Transwell assay for WRO and FRO cells. One-way analysis of variance (ANOVA) and Welch's ANOVA were applied for quantitative analyses. The results shown are representative of three independent experiments. ∗*P* < 0.05; ∗∗*P* < 0.01; ∗∗∗*P* < 0.001; ∗∗∗∗*P* < 0.0001.

**Figure S5:**
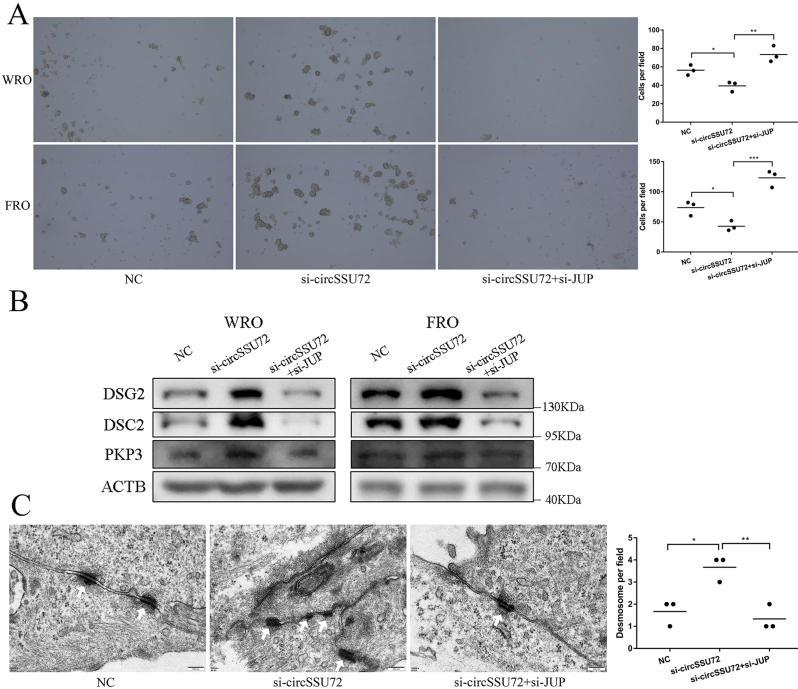
The circSSU72/JUP axis affects desmosomes in thyroid cancer cells. **(A)** Representative images and quantitative analysis of the hanging-drop assay. **(B)** The desmosome-related proteins were examined with the regulation of the circSSU72/JUP axis. **(C)** Representative images and quantitative analysis of transmission electron microscopy. One-way analysis of variance (ANOVA) and Welch's ANOVA were applied for quantitative analyses. The results shown are representative of three independent experiments. ∗*P* < 0.05; ∗∗*P* < 0.01; ∗∗∗*P* < 0.001; ∗∗∗∗*P* < 0.0001.

**Figure S6:**
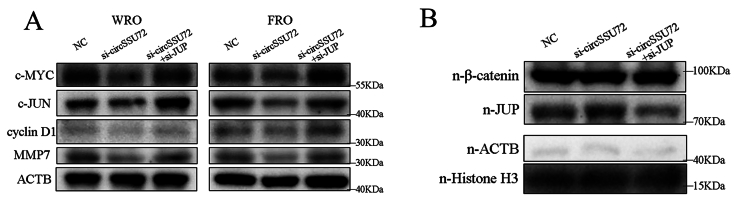
The circSSU72/JUP axis affected the beta-catenin signaling pathway. **(A)** The beta-catenin signaling pathway was activated by the circSSU72/JUP axis. **(B)** The expression of beta-catenin and JUP in the nucleus.

**Figure S7:**
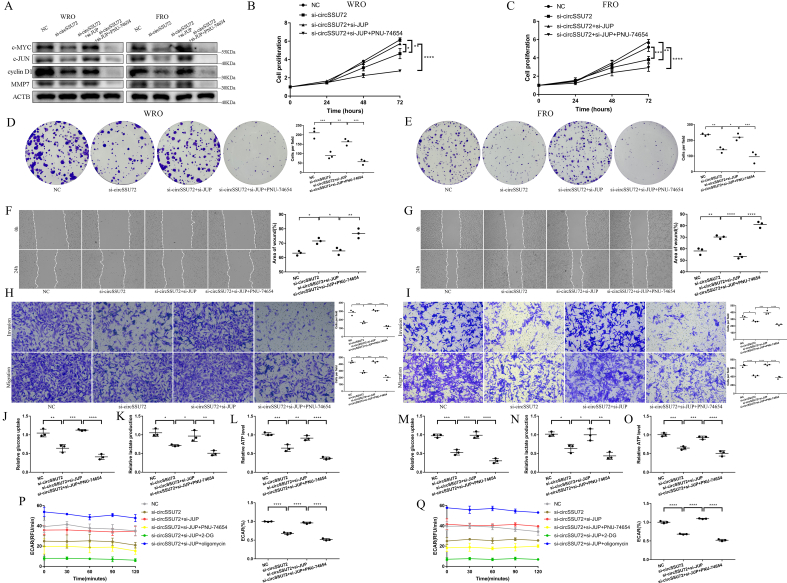
The circSSU72/JUP axis affected thyroid cancer cell proliferation, migration, invasion, and glycolysis via the beta-catenin signaling pathway. **(A)** Activation of the beta-catenin signaling pathway by the circSSU72/JUP axis was inhibited by 20 μM PNU-74654 (a beta-catenin signaling pathway inhibitor). **(B, C)** CCK-8 assay for WRO and FRO cells. **(D, E)** Representative images and quantitative analysis of colony formation in WRO and FRO cells. **(F, G)** Representative images and quantitative analysis of the wound healing assay for WRO and FRO cells. **(H, I)** Representative images and quantitative analysis of the Transwell assay for WRO and FRO cells. **(J**–**L)** Glucose uptake, lactate production, and ATP levels were examined in WRO cells (*n* = 3). **(M**–**O)** Glucose uptake, lactate production, and ATP levels were examined in FRO cells (*n* = 3). **(P, Q)** The extracellular acidification rate was examined in WRO and FRO cells (*n* = 3). One-way analysis of variance (ANOVA) and Welch's ANOVA were applied for quantitative analyses. The results shown are representative of three independent experiments. ∗*P* < 0.05; ∗∗*P* < 0.01; ∗∗∗*P* < 0.001; ∗∗∗∗*P* < 0.0001.

**Figure S8:**
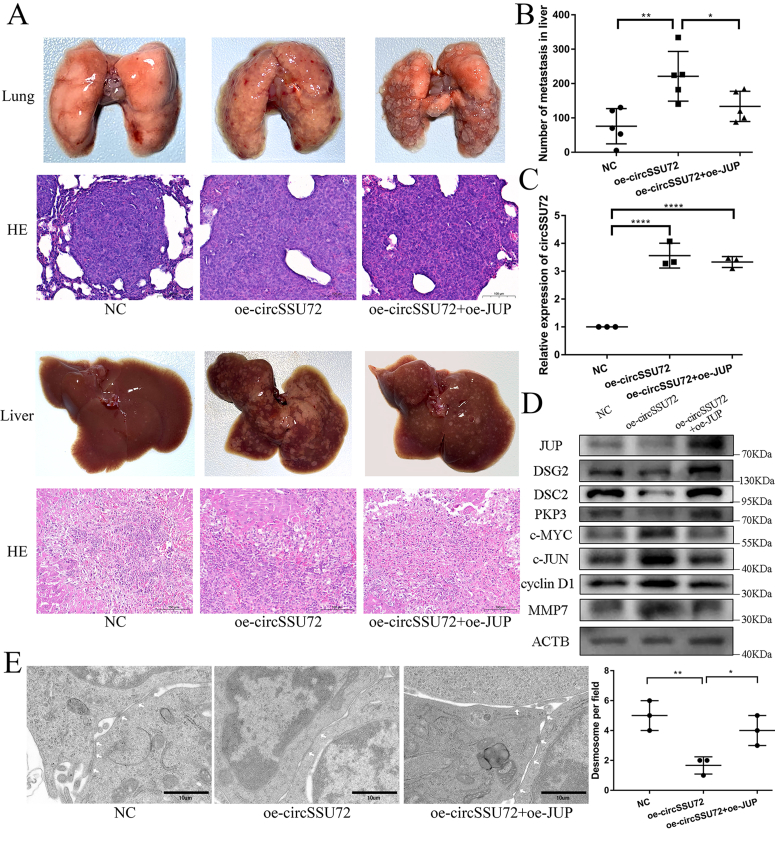
circSSU72/JUP axis affected thyroid cancer metastasis *in vivo*. **(A, B)** Representative images of lung and liver metastasis in nude mice and quantitative analysis of the number of metastatic tumors (*n* = 5). **(C)** Validation of circSSU72 overexpression by quantitative reverse transcription PCR. **(D)** The expression of proteins related to desmosomes and the beta-catenin signaling pathway. **(E)** Representative images and quantitative analysis of the transmission electron microscopy. One-way analysis of variance (ANOVA) and Welch's ANOVA were applied for quantitative analyses. ∗*P* < 0.05; ∗∗*P* < 0.01; ∗∗∗*P* < 0.001; ∗∗∗∗*P* < 0.0001.

**Figure S9:**
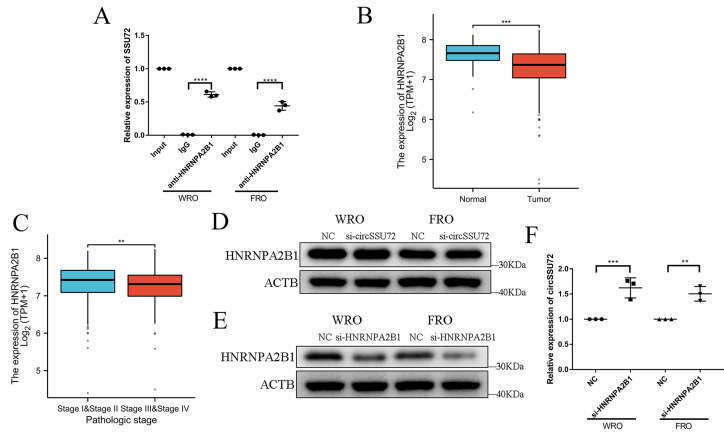
m^6^A modification was involved in the up-regulated level of circSSU72. **(A)** RNA immunoprecipitation confirmed the interaction between linear SSU72 mRNA and HNRNPA2B1 (*n* = 3). **(B)** In the TCGA-THCA dataset, HNRNPA2B1 was significantly down-regulated in thyroid cancer tissues. **(C)** HNRNPA2B1 was associated with the advanced tumor stage in the TCGA-THCA dataset. **(D)** The knockdown of circSSU72 showed no effect on the expression level of HNRNPA2B1. **(E, F)** The knockdown of HNRNPA2B1 increased the expression level of circSSU72. One-way analysis of variance (ANOVA) and Welch's ANOVA were applied for quantitative analyses. ∗*P* < 0.05; ∗∗*P* < 0.01; ∗∗∗*P* < 0.001; ∗∗∗∗*P* < 0.0001.
