# Supplementary material for: Epigenetic modulation of thyroid cancer metastasis and glycolysis through circSSU72-mediated ubiquitination of gamma-catenin and beta-catenin signaling
Source: Genes Dis. 2024 Dec 7;12(4):101485. doi: 10.1016/j.gendis.2024.101485 (PMC11995063; doi:10.1016/j.gendis.2024.101485)
Supplement: Multimedia component 1 [file mmc1.docx]

**Patient samples**

Twenty-six pairs of PTC tissues, 6 pairs of FTC tissues were obtained from TC patients undergoing thyroidectomy at Xiangya Hospital, Central South University. The diagnosis, capsule invasion and lymph node metastasis were determined by postoperative pathology. The exclusion criteria were as follows: (1) under the age of 18; (2) previously received thyroidectomy; (3) suffering from infectious diseases or parathyroid disease; (4) lack of essential clinical data.

**Cell culture**

Human thyroid normal epithelial cell line (Nthy-ori 3-1), human papillary thyroid carcinoma (PTC) cell lines (IHH-4, TPC-1), human follicular thyroid carcinoma (FTC) cell line (WRO), human anaplastic thyroid cancer (ATC) cell lines (8305C, BHT-101, CAL-62, FRO), and human embryonic kidney cell line (HEK293T) were acquired from the Shanghai Academy of Sciences and used in this study. Dulbecco’s modified Eagle’s medium (DMEM) was used to culture Nthy-ori 3-1 and HEK293T cell lines, while RPMI-1640 medium (Gibco, USA) was used to culture other cell lines.

**RNA extraction and quantitative real‑time PCR (RT‑qPCR) assay**

RNAEX reagent (Accurate Biotechnology, Hunan) was used in this study to extract total RNA according to the manufacturer’s instructions. An Evo M-MLV Mix Kit and SYBR Green Premix Pro Taq HS qPCR Kit (Accurate Biotechnology, Hunan) were used for RT‒qPCR assays on a QuantStudio 5 system (Thermo Fisher Scientific, USA) according to the manufacturer’s instructions. The relative expression levels were normalized to ACTB.

**Fluorescence in situ hybridization (FISH) assay**

FISH was performed using a FISH kit (RiboBio, Guangzhou, China). Cells were cultured in a 24-well plate. RNA localization was determined with circSSU72 back-splicing-specific probes (red), and the nucleus was stained by DAPI.

**RNA stability assay**

Actinomycin D was used to determine the RNA decay rate. Cells were treated with actinomycin D (10 µg/ml) in the incubator and harvested for RT-PCR using the method above at 0, 4, 8, 12 hours. The RNA decay rate was calculated using the Ct value.

**Oligonucleotide and stable transfection**

The hsa_circ_0009294 siRNAs, JUP siRNAs, HNRNPA2B1 siRNAs, and negative control (NC) were obtained from Sangon (Shanghai, China). Lipo8000 (Beyotime, Shanghai, China) was used for subsequent transfection according to the manufacturer’s instructions. For each well in a 6-well plate, 4ul Lipo8000 and 100pmol siRNA were used for 24 or 48 hours transfection. For stable transfection, human lentivirus-hsa_circ_0009294 and lentivirus-JUP were obtained from Genechem (Shanghai, China). For each well in a 6-well plate, 40ul HitransG P (Genechem) and 2*10^6^ TU lentivirus were used for 16 hours transfection, and puromycin (Gibco, USA) was used for subsequent cell selection.

**Western blot analysis**

RIPA buffer (Beyotime, Shanghai, China) was used to extract protein, and a Nuclear and Cytoplasmic Protein Extraction Kit (Beyotime, Shanghai, China) was used to extract nuclear protein and cytoplasmic protein separately. After determining the protein content by the BCA method (Thermo Fisher Scientific), an equivalent amount of protein was separated by SDS‒PAGE and transferred to a polyvinyl fluoride membrane. The immune response bands were exposed by Image Quant 800 (Amasia Biotechnology, China). The primary antibodies involved in the present study were anti-JUP (1:1000, Cell Signaling Technology), anti-ACTB (1:1000, Proteintech), anti-Flag (1:1000, Cell Signaling Technology), anti-Ubiquitin (1:1000, Cell Signaling Technology), anti-DSG2 (1:1000, Proteintech), anti-DSC2 (1:1000, Proteintech), anti-PKP3 (1:1000, Proteintech), anti-c-MYC (1:1000, Cell Signaling Technology), anti-c-JUN (1:1000, Proteintech), anti-cyclin D1 (1:1000, Proteintech), anti-MMP7 (1:1000, Proteintech), and anti-HNRNPA2B1 (1:1000, Cell Signaling Technology).

**Cell proliferation analysis**

Cell Counting Kit-8 (Beyotime, Shanghai, China) and colony formation assays were used to detect cell proliferation ability. Equivalent cells were seeded in 96-well plates, and 10 µl CCK-8 solution was added to each well at different time points. After further incubation for 2 h, the absorbance at 450 nm was measured on a microplate reader. For the colony formation assay, cells were seeded in 6-well plates and cultured for 10 days in a cell incubator, and colonies were stained with 0.1% crystal violet.

**Wound healing assay**

Cells were cultured in 6-well plates at 37 °C. Wounds were vertically created with 100 μl pipette tips. The scratched cells were removed with PBS, and 200 µl medium without FBS was added to each well. Images of the wound were observed and photographed at 0 and 24 hours.

**Transwell assay**

Polycarbonate transwell filters (Corning, USA) were used to perform a transwell assay. The same number of cells in FBS-free medium were seeded in upper chambers with or without Matrigel (Corning, USA). The migrated or invaded cells were counted under a microscope.

**Hanging-drop assay**

A single-cell suspension of 3000 cells in 20 µl of medium was suspended as a hanging drop from the lid of a 24-well culture dish, and 2 ml of PBS was added to each well to avoid evaporation. After 24 hours of culturing at 37 °C, the single cells were observed as the loss of aggregation ability and counted by microscopy.

**Glucose uptake assay**

A glucose uptake assay kit (Abcam, UK) was used to perform the glucose uptake assay. Briefly, 8000 cells were seeded in 96-well plates and cultured in FBS-free medium overnight. After incubating cells with KRPH buffer for 40 min, 10 µl 2-deoxyglucose was added to each well for a further 20 min incubation. Extraction buffer was used for cell lysis. After centrifuging (500 rpm) for 1-2 min, the supernatants were collected and further processed for absorbance measurement at 412 nm on a microplate reader according to the instructions from the manufacturer.

**Lactate production assay**

A lactate detection assay kit was acquired from Solarbio (Beijing, China). Briefly, equivalent cells were lysed with lysis buffer and ultrasonicated. After centrifuging (12000 g) for 10 min at 4°C, the supernatants were collected and further processed for absorbance measurement at 570 nm on a microplate reader.

**ATP detection assay**

An enhanced ATP assay kit was acquired from Beyotime (Shanghai, China) and used according to the manufacturer’s instructions. Briefly, equivalent cells were lysed in lysis buffer and centrifuged (12000 g) for 5 min at 4°C. The supernatants were collected and further processed for the measurement of luminescence signals on a luminometer.

**Extracellular acidification rate (ECAR) assay**

**The** ECAR assay kit was acquired from Bestbio (Shanghai, China) using a pH-sensitive fluorescent probe. Equivalent cells were seeded in a black 96-well plate. After incubating the cells without CO_2_ for 2 h to exclude interference, the probe was diluted in buffer and then added to each well. Ten microliters of 2-DG (500 mmol/L) was used as a negative control, while 1 µl of oligomycin (5 mM) was used as a positive control. The plate was read every 5 min by a luminometer (Ex 488/Em 580) at 37°C, and the ECAR was calculated as the slope of the kinetic curve according to the instructions from the manufacturer.

**Chromatin isolation by RNA purification & mass spectrometry (ChiRP**-**MS)**

The ChiRP-MS technique was used to study the specific proteins that bind to circSSU72. Briefly, cells were washed with PBS and incubated with lysis buffer. After the extracts were sonicated and centrifuged, the supernatants were collected for further hybridization. Next, cells were hybridized with circSSU72 antisense probes labeled with biotin and bound to magnetic beads, while lacZ was used in the control group, which was obtained from Genechem (Shanghai, China). The bound proteins were eluted for mass spectrometry analysis by liquid chromatography-coupled tandem MS (LC‒MS) using a Thermo Fisher Q Exactive system (Thermo Fisher Scientific, USA). The proteomic analysis was performed based on the UniProt database.

**RNA immunoprecipitation (RIP)**

The binding between RNA and protein was further confirmed by RIP assay using the RIP kit (BersinBio, China) according to the manufacturer’s instructions. In short, cells were washed with PBS and incubated with lysis buffer. DNase was used to digest DNA, and the supernatant was collected after the extracts were centrifuged for 10 min at 4°C. The supernatant was subsequently incubated with magnetic beads conjugated to anti-JUP antibody, anti-DESP antibody, anti-RBBP6 antibody, anti-HNRNPA2B1 antibody, anti-m6A antibody, or anti-IgG antibody. RNA was extracted and examined by RT-qPCR using the method we mentioned above.

**Immunoprecipitation (IP)**

An immunoprecipitation kit (Beyotime, China) and protein A+G magnetic beads (Beyotime, China) were used for the IP assay. Cells were lysed with lysis buffer and incubated with specific antibodies overnight at 4°C. The magnetic beads were added to the cell lysates for a further 2 h of incubation and were subsequently washed and boiled for 5 min at 95°C. The supernatants were used for western blot assays.

**Cycloheximide chase assays**

To investigate the stability of proteins, cells were seeded into 6-well plates and treated with cycloheximide (CHX, Selleck, USA) at a final concentration of 200 μM when 70% confluence was achieved. Subsequently, the proteins were extracted from these cells at different time points for WB as shown above.

**Transmission electron microscopy (TEM)**

For ultrastructural studies, cells and tissues were fixed in 2.5% glutaraldehyde followed by 1% osmium tetroxide. After gradient dehydration, cells and tissues were embedded, ultrathin-sectioned (70 nm), stained, and observed under an HT7700 transmission electron microscope (Hitachi, Japan). The desmosome numbers were quantified and used for comparisons. Specifically, we adopted in situ embedding on the TEM of monolayer cells to maintain the integrity of desmosomes.^13^

**Chromatin immunoprecipitation (ChIP)**

ChIP assays were performed using a BeyoChIP ChIP Assay Kit (Beyotime, Shanghai, China) according to the manufacturer’s instructions. Briefly, adequate cells were collected, followed by cross-linking, uncrosslinking, and sonication fragmentation. The chromatin fragments were immunoprecipitated by specific antibodies with the IgG antibody as a control. qPCR was used to detect certain extracted DNA.

**Dual-luciferase assay**

The TOP firefly luciferase reporter plasmid, renilla luciferase reporter plasmid, and Dual Luciferase Reporter Gene Assay Kit were obtained from Beyotime Corp. (Shanghai, China). Plasmids and siRNAs were cotransfected for 48 hours with Lipo8000 (Beyotime, Shanghai, China) according to the manufacturer’s instructions. Relative luciferase activity was normalized to renilla luciferase activity.

**Metastasis animal model**

Six-week-old male BALB/c nude mice were purchased from the Department of Laboratory Animal Science, Central South University. A total of 10^7^ cells were injected into the tail vein to establish metastasis models (n = 5). After 8 weeks, pulmonary and liver metastases were confirmed by hematoxylin and eosin (HE) staining. The number of metastases was counted and used for comparisons, and tumor tissues were obtained for further experiments. All animal experiments were approved by the Ethics Committee of Xiangya Hospital.

**Statistical analyses**

Statistical Package for Social Sciences 23.0 for Windows (SPSS Inc., USA) and GraphPad Prism v7.0 software (GraphPad Software, USA) were used to perform statistical analyses and generate illustrations. One-way analysis of variance (ANOVA) was used for homogeneous variance, while Welch’s ANOVA was applied when the variance was heterogeneous. A P value < 0.05 was considered statistically significant.

The sequence of primers involved in this study.

| Gene | Sequence (5’-3’) | |
| --- | --- | --- |
| circSSU72 | Forward | GTGTGCACTTCCCGACATAC |
|  | Reverse | GGAATTCAGATTGACAGCAGCA |
| SSU72 | Forward | CGACAAGCCCAATGTTTATGAT |
|  | Reverse | ATCAAACAGGTCTTTGCAGTTC |
| JUP | Forward | TGGAGGTGCTGGATAGGCTATGG |
|  | Reverse | AGGAGGCAGAGGACTCAAGAATGG |
| ACTB | Forward | CCTGGCACCCAGCACAAT |
|  | Reverse | GGGCCGGACTCGTCATAC |
| MYC-enhancer | Forward | AGGCAACCTCCCTCTCGCCCTA |
|  | Reverse | AGCAGCAGATACCGCCCCTCCT |

RT, reverse transcription.
