# Supplementary material for: Epigenetic modulation of thyroid cancer metastasis and glycolysis through circSSU72-mediated ubiquitination of gamma-catenin and beta-catenin signaling
Source: Genes Dis. 2024 Dec 7;12(4):101485. doi: 10.1016/j.gendis.2024.101485 (PMC11995063; doi:10.1016/j.gendis.2024.101485)
Supplement: Multimedia component 3 [file mmc3.docx]

**Supplement Table 1**. The expression of circSSU72 correlates with tumor characteristics

| Characteristics | circSSU72-low group (n=16) | circSSU72-high group (n=16) | *P* |
| --- | --- | --- | --- |
| Age (years) | 39.19±6.98 | 37.13±7.06 | 0.413 |
| Gender |  |  | 0.458 |
| Female | 12 (75.0) | 9 (56.3) |  |
| Male | 4 (25.0) | 7 (43.7) |  |
| Bilateral lesion |  |  | 0.704 |
| Yes | 3 (81.3) | 7 (43.7) |  |
| No | 13 (18.7) | 9 (56.3) |  |
| Largest tumor size (cm) | 0.66±0.59 | 1.04±0.39 | 0.040 |
| Number of lesion |  |  | 0.066 |
| Single | 13 (81.3) | 7 (43.7) |  |
| Multiple | 3 (18.7) | 9 (56.3) |  |
| Capsule invasion |  |  | 0.043 |
| Yes | 0 (0.0) | 5 (31.3) |  |
| No | 16 (100.0) | 11 (68.7) |  |
| Lymph node metastasis |  |  | 0.003 |
| Yes | 5 (31.3) | 14 (87.5) |  |
| No | 11 (68.7) | 2 (12.5) |  |
| JUP expression level (IHC) | 4.63±0.46 | 3.19±0.44 | 0.032 |

Data are expressed as mean±standard deviation or n (%). Chi-square, Fisher exact test and Welch’s t test were applied for quantitative analyses.
