# Supplementary material for: Epigenetic modulation of thyroid cancer metastasis and glycolysis through circSSU72-mediated ubiquitination of gamma-catenin and beta-catenin signaling
Source: Genes Dis. 2024 Dec 7;12(4):101485. doi: 10.1016/j.gendis.2024.101485 (PMC11995063; doi:10.1016/j.gendis.2024.101485)
Supplement: Multimedia component 4 [file mmc4.docx]

**Supplement Table 2**. The expression of JUP correlates with tumor characteristics

| Characteristics | JUP-low group (n=16) | JUP-high group (n=16) | *P* |
| --- | --- | --- | --- |
| Age (years) | 37.56±7.15 | 38.75±7.00 | 0.638 |
| Gender |  |  | 1.000 |
| Female | 11 (68.7) | 10 (62.5) |  |
| Male | 5 (31.3) | 6 (37.5) |  |
| Bilateral lesion |  |  | 0.252 |
| Yes | 7 (43.7) | 3 (81.3) |  |
| No | 9 (56.3) | 13 (18.7) |  |
| Largest tumor size (cm) | 0.96±0.44 | 0.74±0.60 | 0.250 |
| Number of lesion |  |  | 0.716 |
| Single | 9 (56.3) | 11 (68.7) |  |
| Multiple | 7 (43.7) | 5 (31.3) |  |
| Capsule invasion |  |  | 0.333 |
| Yes | 4 (25.0) | 1 (6.3) |  |
| No | 12 (75.0) | 15 (93.7) |  |
| Lymph node metastasis |  |  | 0.029 |
| Yes | 13 (81.3) | 10 (62.5) |  |
| No | 3 (18.7) | 6 (37.5) |  |

Data are expressed as mean±standard deviation or n (%). Chi-square, Fisher exact test and Welch’s t test were applied for quantitative analyses.
